# Functional Characterization of a Sugar Beet *BvbHLH93* Transcription Factor in Salt Stress Tolerance

**DOI:** 10.3390/ijms22073669

**Published:** 2021-04-01

**Authors:** Yuguang Wang, Shuang Wang, Ye Tian, Qiuhong Wang, Sixue Chen, Hongli Li, Chunquan Ma, Haiying Li

**Affiliations:** 1 Engineering Research Center of Agricultural Microbiology Technology, Ministry of Education, Heilongjiang University, Harbin 150080, China; wangyuguang@hlju.edu.cn (Y.W.); laissezfairewk@gmail.com (S.W.); ty8259660@sina.cn (Y.T.); lihongli2082@hlju.edu.cn (H.L.); 2 National Sugar Crop Improvement Centre, College of Advanced Agriculture and Ecological Environment, Heilongjiang University, Harbin 150080, China; wangqiuhong@hlju.edu.cn; 3 Key Laboratory of Molecular Biology of Heilongjiang Province, College of Life Sciences, Heilongjiang University, Harbin 150080, China; 4 Plant Molecular and Cellular Biology Program, Department of Biology, Genetics Institute, University of Florida, Gainesville, FL 32610, USA; schen@ufl.edu

**Keywords:** sugar beet, salt stress, bHLH, transcription factor, antioxidant enzymes

## Abstract

The basic/helix–loop–helix (bHLH) transcription factor (TF) plays an important role for plant growth, development, and stress responses. Previously, proteomics of NaCl treated sugar beet leaves revealed that a bHLH TF, *BvbHLH93*, was significantly increased under salt stress. The BvbHLH93 protein localized in the nucleus and exhibited activation activity. The expression of *BvbHLH93* was significantly up-regulated in roots and leaves by salt stress, and the highest expression level in roots and leaves was 24 and 48 h after salt stress, respectively. Furthermore, constitutive expression of *BvbHLH93* conferred enhanced salt tolerance in *Arabidopsis,* as indicated by longer roots and higher content of chlorophyll than wild type. Additionally, the ectopic expression lines accumulated less Na^+^ and MDA, but more K^+^ than the WT. Overexpression of the *BvBHLH93* enhanced the activities of antioxidant enzymes by positively regulating the expression of antioxidant genes *SOD* and *POD*. Compared to WT, the overexpression plants also had low expression levels of *RbohD* and *RbohF*, which are involved in reactive oxygen species (ROS) production. These results suggest that *BvbHLH93* plays a key role in enhancing salt stress tolerance by enhancing antioxidant enzymes and decreasing ROS generation.

## 1. Introduction

Plants frequently encounter adverse environmental conditions during their growth and development, such as pathogen, drought, high temperature, cold, and salt stress. Salinity is a major environmental threat for crop production, because a high concentration of salt in the soil severely affects plant performance by causing metabolic perturbation, ion toxicity, and hyperosmotic stress [1]. In addition, salt stress can also lead to secondary stress such as oxidative stress [2]. In order to maintain normal growth under salt stress, plants have developed complex mechanisms to respond and adapt to high salt environments. The adaptive responses are coordinated by regulating related gene expression. Transcription factors (TFs) play a key role in transcriptional control by activating or repressing their related downstream genes [3]. Numerous transcription factors, including MYB (v-myb avian myeloblastosis viral oncogene), ERF (ethylene responsive element binding factor), bHLH (basic helix–loop–helix), bZIP (basic region/leucine zipper), DREB (dehydration responsive element-binding), and WRKY (“WRKY” four conserved amino acid sequences) families, were found to mediate plant response to salt stress [4].

In plants, the bHLH family is the second largest class of plant TFs involved in diverse biological and metabolic processes important for plant growth, development, and response to environmental changes [5]. The bHLH domains are highly conserved with two functionally distinct regions (the basic and HLH regions). The basic region (at the *N*-terminus) contains 13–17 basic amino acids generally, which are involved in DNA binding. The HLH region is distributed at the N-terminus of the bHLH conserved domain, which is related to the formation of homologous or heterologous dimers of the bHLH TFs [6]. Recently, several studies have found that bHLH TFs are involved in regulating plant salt tolerance through regulating gene expression. Overexpression of a bHLH gene (*OrbHLH001*) from *Oryza rufipogon* confers freezing and salt tolerance in transgenic *Arabidopsis* [7]. In *Arabidopsis*, bHLH122 functions as a positive regulator of NaCl and osmotic stress signaling. Transgenic plants overexpressing *bHLH122* displayed great resistance to NaCl stress. Moreover, bHLH122 could bind directly to the G-box/E-box cis-elements in the *cytochrome P450* (*CYP707A3*) promoter to repress its expression [8]. Although several bHLHs related to salt stress tolerance have been identified in *Arabidopsis*, rice, and wheat [9,10,11,12], there are few studies on their involvement in regulating salt tolerance in sugar beet (*Beta vulgaris* L.).

Sugar beet, a species of *Chenopodiaceae* family, is an important sugar crop in the world [13]. It inherited salt-tolerance traits from *Beta maritima* L., which is a wild ancestor of sugar beet [14]. Sugar beet can tolerate up to 500 mM sodium chloride (NaCl) for seven days without losing viability [15]. Moreover, it has been found that when the electrical conductivity (EC) of soil reached 7.0 dS·m^−1^, the yield of sugar beet was not affected [13], but it is sensitive to salt stress at germination and seedling stages. Therefore, the understanding of molecular and physiological mechanisms of sugar beet salt tolerance will help to develop sugar beet cultivars with high salinity tolerance. However, insufficient information is available regarding salt tolerance-related genes and the mechanisms in the sugar beet. In particular, the role of bHLHs in regulating salt tolerance in sugar beet has not been reported. In this study, we report the identification of a bHLH TF *BvbHLH93* as a salt responsive gene in sugar beet, and the characterization of the BvbHLH93 in mediating stress responses, resulting in improved plant salt stress tolerance.

## 2. Results

### 2.1. Isolation of BvbHLH93 and Sequence Analysis

**Figure 1:**
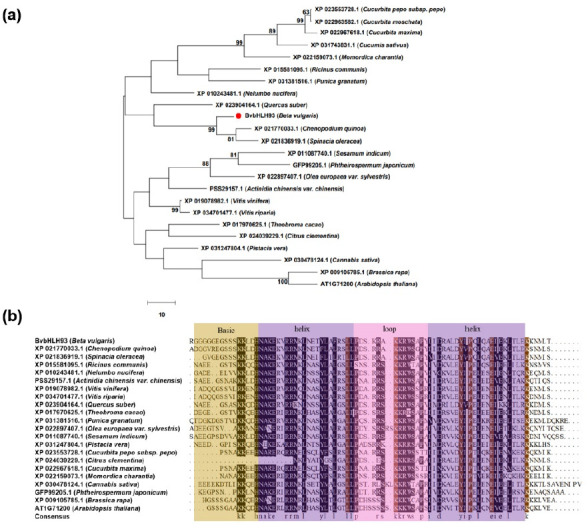
Phylogenetic relationship and sequence alignment of the BvbHLH93 and its orthologs from other plants. (**a**) The phylogenetic tree was based on the full-length protein sequences of BvbHLH93 and its orthologs from other plants. (**b**) Sequence alignment of the deduced amino acid sequences of BvbHLH93 and its orthologs from other plants.

Previously, we used iTRAQ-based proteomics to profile protein changes in sugar beet (*Beta vulgaris* L.) T510 under salt stress. We found that *BvbHLH93* (*LOC104894793*) was strongly induced by salt stress. The *BvbHLH93* is 4101 bp spanning three exons and two introns located on chromosome 5. The full-length *BvbHLH93* cDNA sequence of 1516 bp was amplified by RT-PCR (Figure S1). Analysis of the sequence illustrated that the *BvbHLH93* ORF sequence contained 1014 bp and encoded a protein of 337 amino acid residues (Figure S1), with a calculated molecular mass of 37.8 kDa and a PI of 5.19. Several plant BvbHLH93 homologs present in the NCBI non-redundant database were identified by BLASTP search using the BvbHLH93 sequence as query. Phylogenetic analysis based on amino acid sequence of BvbHLH93 and homologs from other species revealed that the *Bv*bHLH93 protein has the closest homology relationship with two bHLH proteins in *Spinacia oleracea* and *Chenopodium quinoa* (Figure 1a). Multiple sequence alignment of deduced amino acid sequences of these proteins revealed that the BvbHLH93 had a conservative bHLH domain shared by other bHLH homologs (Figure 1b).

### 2.2. Subcellular Localization and Transcriptional Activating of the BvbHLH93 Protein

**Figure 2:**
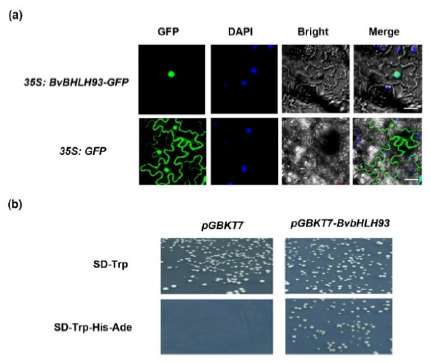
Subcellular localization and activation activity analysis of the BvbHLH93. (**a**) Subcellular localization of BvbHLH93 in *Nicotiana tabacum* L. (Bar = 50 μm). (**b**) Activation activity of BvbHLH93. The transformants with pGBKT7 (left) and pGBKT7-*BvBHLH9*3 (right) grow normally on SD-Trp medium, and only the transformants pGBKT7-*BvBHLH93* (right) can grow normally on SD-Trp-His-Ade medium.

To examine the subcellular localization of BvbHLH93 protein, the open reading frame (ORF) of *BvbHLH93* sequence was fused to the Green fluorescent protein (GFP) gene sequence in the pCAMBIA2300-GFP vector. Recombinant constructs pCAMBIA2300-*BvbHLH93-*GFP and pCAMBIA2300-GFP vectors were transiently expressed in *Nicotiana benthamiana* leaves, respectively. The results revealed the BvbHLH93-GFP fluorescence was only in the nuclei, whereas the fluorescence of GFP alone was distributed throughout the whole cell (Figure 2a). Furthermore, the nuclear dihydrochloride (DAPI) staining also confirmed the nuclear localization of BvbHLH93.

To further investigate whether BvbHLH93 is a transcriptional factor, the activation activity of BvbHLH93 was determined. pGBKT7-*BvbHLH93* was constructed to test if BvbHLH93 can activate reporter genes *His3* and *Ade2* in *AH109*. The yeast transformants containing either pGBKT7 (negative control) or pGBKT7-*BvbHLH93* grew normally on SD/-Trp medium. However, only the yeast transformed with pGBKT7-*BvbHLH93* grew normally on SD-Trp-His-Ade medium (Figure 2b). These results indicate that BvbHLH93 activated the transcription of the *His3* and *Ade2* reporter genes and acted as a transcriptional activator.

### 2.3. Stress Responses of BvbHLH93 Transcription

**Figure 3:**
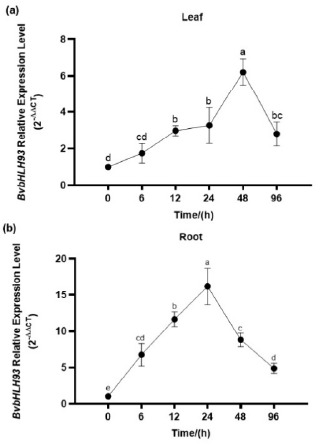
*BvbHLH93* expression patterns in response to salt stress treatments. (**a**) Leaves and (**b**) roots of the sugar beet plants treated with 200 mM NaCl for different time periods. Data are the means of three biological replicates with standard deviation (SD) bars. Different letters indicate significant difference at *p* < 0.05.

To investigate the functions of the *BvbHLH93* in regulating plant responses to salt stress, we examined the expression of *BvbHLH93* in response to salt stress by using qRT-PCR (Figure 3). *BvbHLH93* expression was significantly up-regulated in roots and leaves by salt stress. After salt stress, the increase of *BvbHLH93* transcript level appeared much earlier in roots than in leaves, and the highest expression level in roots and leaves was at 24 and 48 h, respectively (Figure 3). These results indicate that the *BvbHLH93* transcription was significantly induced in roots and leaves by salt stress.

### 2.4. Overexpression of BvbHLH93 Increased Salt Stress Tolerance in Arabidopsis

**Figure 4:**
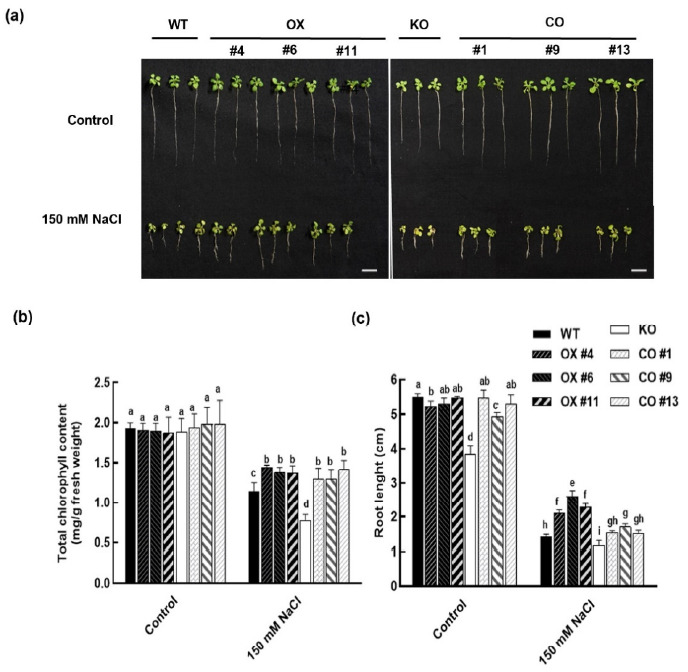
Effects of salt stress on seedling growth phenotype, chlorophyll, and root length in wild type (WT), *BvbHLH93*-overexpression in wild type *Arabidopsis* (OX), *atbhlh93* knock-out mutant (KO), and transgenic *BvbHLH93* in the KO mutant (CO). (**a**) Eight-day-old seedlings were transferred to MS medium containing 150 mM NaCl for seven days. (**b**) Chlorophyll level and (**c**) root length in control and 150 mM NaCl treated seedlings. Different letters indicate significant difference at *p* < 0.05. Three biological replicates were performed.

The salt-inducible expression of *BvbHLH93* in roots and leaves prompted us to analyze its potential role in salt resistance. Three independent overexpression *Arabidopsis* lines (OX4, OX6, and OX11) were selected for analysis based on the *BvbHLH93* expression levels (Figure S2a). BLASTp analysis of the BvbHLH93 protein sequences in the *Arabidopsis* database showed that the BvbHLH93 had the highest sequence similarity to an *Arabidopsis* BHLH protein (*At1g71200*). Therefore, a T-DNA insertion mutant of *At1g71200* was acquired (SALK_073160), and the expression of this gene in the mutant was determined (Figure S2b). Furthermore, we transformed the knock-out (KO) mutant with the *BvbHLH93* overexpression vector to develop homozygous T3 complementation transgenic lines (CO1, CO9, and CO13). Under normal growth conditions, there were no differences in growth phenotype among these plants, except for mutant plants, whose root growth was slower than the WT plants (Figure 4). Under salt stress, the three OX lines had significantly longer roots and higher chlorophyll contents than WT and the KO lines (Figure 4a–c), and the contents of chlorophyll in the KO lines were significantly lower than WT plants and the CO lines (Figure 4c). Collectively, these results show that the *BvbHLH93* is involved in regulating plant salt stress response, and overexpression of *BvbHLH93* conferred salt stress tolerance.

**Figure 5:**
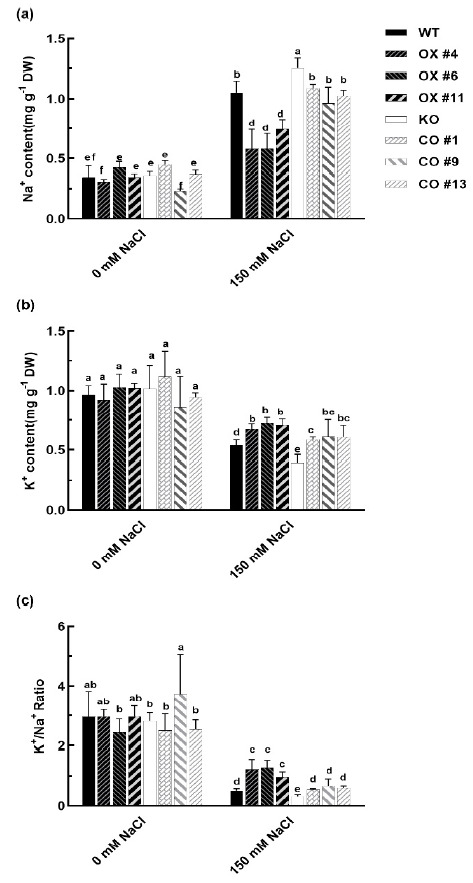
Na+ and K+ contents in the leaves of wild type (WT), *BvbHLH93*-overexpression *Arabidopsis* (OX), *atbhlh93* mutant (KO), and transgenic *BvbHLH93* in the mutant (CO) under salt stress. (**a**) Na^+^ content of the leaves. (**b**) K^+^ content of the leaves. (**c**) K^+^ to Na^+^ ratio of the leaves. Different letters indicate significant difference at *p* < 0.05. Three biological replicates were performed.

In order to determine the K^+^/Na^+^ ratio in plants, we analyzed the ion contents of WT, OX, KO, and CO plants under normal or salt stress conditions (Figure 5). Without NaCl stress, Na^+^ contents were similar in WT, OX, and KO lines (Figure 5a). Under NaCl treatment, Na^+^ levels increased in all the plants, but the shoot Na^+^ contents in the OX lines were significantly lower than the WT lines. The Na^+^ contents in the KO lines were significantly higher than the WT and CO lines (Figure 5a). Moreover, K^+^ levels decreased under salt stress in WT, OX, KO, and CO lines; the extent of decrease was significantly lower in the shoots of OX lines than the WT and mutant lines (Figure 5b). Furthermore, the OX lines maintained significantly higher K^+^/ Na^+^ ratios than the WT and mutant under salt stress (Figure 5c). It is well-established that salt-tolerant plants have more K^+^ and less Na^+^ to maintain a higher K^+^/Na^+^ ratio. These results indicated that BvbHLH93 can regulate ion homeostasis and improve salt tolerance.

### 2.5. BvbHLH93 Enhanced Antioxidative Activities in Arabidopsis

**Figure 6:**
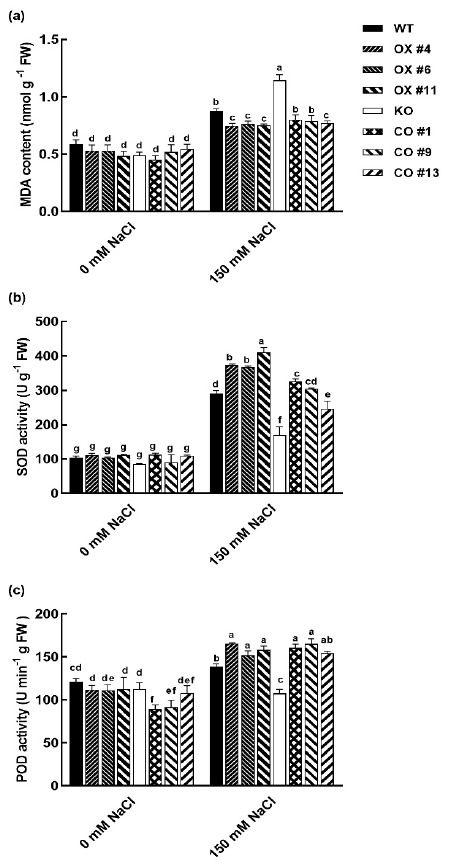
Effects of salt stress on the antioxidant enzyme system in the leaves of wild type (WT), *BvbHLH93*-overexpression *Arabidopsis* (OX), *atbhlh93* mutant (KO), and transgenic *BvbHLH93* in the mutant (CO). (**a**) Leaf malondialdehyde (MDA) content. (**b**) SOD activity. (**c**) POD activity. Different letters indicate significant difference at *p* < 0.05. Three biological replicates were performed.

To examine how *BvbHLH93* affects the plant salt stress tolerance, malondialdehyde (MDA) content and antioxidant activities were determined. As shown in Figure 6, salt stress induced a significant increase in the MDA concentration in all the plant lines, but the MDA level was dramatically higher in the KO mutant than the WT and CO plants (Figure 6a). The OX plants exhibited lower MDA contents than the WT and KO line under salt stress (Figure 6a). Our results indicate that overexpression of *BvbHLH93* reduced the level of lipid peroxidation, which is tightly regulated by enzymes involved in reactive oxygen species (ROS)-detoxifying pathways. Consistently, after salt stress treatment, peroxidase (POD) and superoxide dismutase (SOD) activities were highest in the three OX lines, and lowest in the KO lines (Figure 6b,c). Therefore, we reason that the elevated POD and SOD activities in *BvbHLH93* overexpression lines may result in decreased ROS levels, preventing membrane damage and thus increasing plant salt tolerance.

### 2.6. BvbHLH93 Regulating the Expression of Antioxidant-Related Gene in Arabidopsis

**Figure 7:**
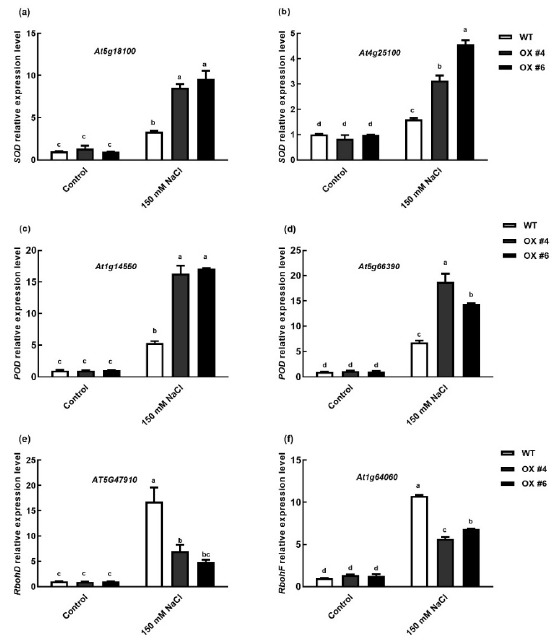
Effects of salt stress on the mRNA levels of *SOD*, *POD*, *RbohD,* and *RbohF* in the leaves of wild type (WT), *BvbHLH93*-overexpression *Arabidopsis* (OX), *atbhlh93* mutant (KO), and transgenic *BvbHLH93* in the mutant (CO). (**a**,**b**) mRNA levels of *SOD* under control and salt stress (150 mM NaCl) conditions. (**c**,**d**) mRNA levels of *POD* under control and salt stress (150 mM NaCl) conditions. (**e**,**f**) mRNA levels of *RbohD* and *RbohF* under control and salt stress (150 mM NaCl) conditions. Different letters indicate significant difference at *p* < 0.05. Three biological replicates were performed.

As overexpression of *BvbHLH93* enhanced the activities of SOD and POD, we examined if the expression of *SOD* and *POD* genes were altered due to the *BvbHLH93* overexpression in the OX lines. Two *SOD* genes (At5g18100 and At4g25100) and two *POD* genes (At1g14550 and At5g66390) have been identified to be associated with SOD and POD activities, and were selected for expression analysis. Our results showed that the transcripts of *SODs* and *POD*s increased significantly in the OX plants compared with WT plants under salt stress (Figure 7a–d). These results demonstrate that BvbHLH93 increased the activities of SOD and POD by positively regulating the transcriptional expression of *SOD* and *POD* genes. Moreover, overexpression of *BvbHLH93* significantly reduced the level of lipid peroxidation. We next determined whether the expression of ROS generation related genes is affected under salt stress. Generation of ROS may be attributed to the expressions of respiratory burst oxidase homolog genes (*RbohD* and *RbohF*). Here, *RbohD* and RbohF expression was significantly increased in the leaves of WT under salt stress (Figure 7e,f). The salt stress-induced transcription of *RbohD* and *RbohF* was significantly inhibited in the leaves of the OX and CO lines compared to WT (Figure 7e,f). Thus, these results demonstrate that BvbHLH93 positively regulated plant salt tolerance through alleviating ROS damage under salt stress by decreasing the expression of *RbohD* or *RbohF* and enhancing *PODs* or *SODs* expression.

## 3. Discussion

Plants are sessile, and they must effectively cope with salt stress to survive. In plant response to the salt stress, TFs play important roles in regulating complicated gene transcription networks. The bHLH gene family is one of the largest families in plants. In previous studies, it was found that bHLH transcription factors were involved in salt and drought stress tolerance in plants [16,17]. To the best of our knowledge, the roles of bHLH TFs in salt tolerance of sugar beet have not been reported. Recently, *BvbHLH137* was found to be increased in a dose-dependent manner under both salt stress and shock treatments in sugar beet [18]. In this study, the expression of *BvbHLH93* was highly induced in sugar beet exposed to NaCl treatment, indicating that this TF may play an important role in salt stress response and tolerance. Under salt stress, the expression patterns of *BvbHLH93* in leaves and roots exhibited significant difference, indicating different regulatory networks of BvbHLH93 in roots and leaves. Moreover, the increased expression of *BvbHLH93* appeared much earlier in sugar beet roots than in leaves, suggesting early salt stress sensing and rapid activation of the *BvbHLH93* in the sugar beet roots. Generally, the level of photosynthetic pigment is closely related to plant salt tolerance [19]. In this study, overexpression of *BvbHLH93* led to increased total chlorophyll content in OX lines compared to WT and mutant plants under salt stress. Thus, the increased chlorophyll content clearly indicates the salt tolerance of the OX plants.

When plants are exposed to a saline environment, Na^+^ can enter cells through non-selective cation channels and K+ transporters. Thus, maintaining ion homeostasis is imperative for plants to adapt to salt stress [20]. In this study, the OX lines maintained significantly higher K^+^/Na^+^ ratios than the WT under salt stress (Figure 5c). These results indicated that BvbHLH93 can regulate ion homeostasis and improve salt tolerance. Plants eliminate excessive Na^+^ from the cytosol via the plasma membrane or tonoplast Na^+^/H^+^ antiporters (NHX) to maintain an optimal cytosolic Na^+^ level. It is reported that the sugar beet *BvNHX1* gene was modulated by MYB transcription factor(s), which were responsible for activating its expression upon salt exposure [21]. Furthermore, plasma membrane (PM) H^+^-ATPase was found to be involved in restricting K^+^ efflux in sugar beet under salt stress conditions [22]. Next, it is important to understand whether BvbHLH93 is involved in the regulation of *NHX* or *H^+^-ATPase* gene expression under salt stress. Recently, BvHb2, a class 2 non-symbiotic hemoglobin, was able to confer drought and osmotic stress tolerance, and BvHb2 was able to confer drought and osmotic stress tolerance involved increasing levels of iron content in leaves [23]. Moreover, an aquaporin gene (*BvCOLD1*) from sugar beet can alter boron homeostasis in yeast and *Arabidopsis* plants. Overexpression of *BvCOLD1* is also able to confer salt stress tolerance to transgenic *Arabidopsis* plants [24]. These studies proved that maintaining ion homeostasis plays an important role in sugar beet salt tolerance, and it is interesting to know whether *BvbHLH93* is involved in mediating the homeostasis of these ions.

MDA contents are proposed to be indicators of oxidative stress. Usually, reducing the use of absorption light energy caused by inhibition of calvin cycle enzyme under stress conditions induces production of ROS, and excessive accumulation of ROS leads to oxidative damage in plants [25,26]. Increased ROS scavenging is a common mechanism to induce stress tolerance in plants. Halophytic plants have evolved an efficient ROS scavenging system [27,28]. For example, SOD dismutates superoxide radicals to H_2_O_2_, which is sequentially scavenged by CAT and POD [29]. In this study, overexpression of *BvbHLH93* was related to reduced MDA levels, suggesting that BvbHLH93 is involved in ROS scavenging. Meanwhile, BvbHLH93 induces an increase in the activities of POD and SOD in sugar beet, mainly through transcriptional activative of *POD* and *SOD* expression in *Arabidopsis*. Thus, constitutive expression of *BvbHLH93* enhanced expression of *POD* and *SOD*, which led to increased antioxidant enzyme activities and salt tolerance. Previously, AtbHLH112 was demonstrated to mediate salt and drought stress tolerance by increasing the expression of *POD* and *SOD* genes to improve ROS scavenging ability [30]. Moreover, overexpression of *ThbHLH1* significantly boosted POD and SOD activities to decrease ROS accumulation [31]. However, whether these transcription factors directly bind to the promoter region of *SOD* and *POD* genes is not known. It is an interesting research direction to pursue in the future.

**Figure 8:**
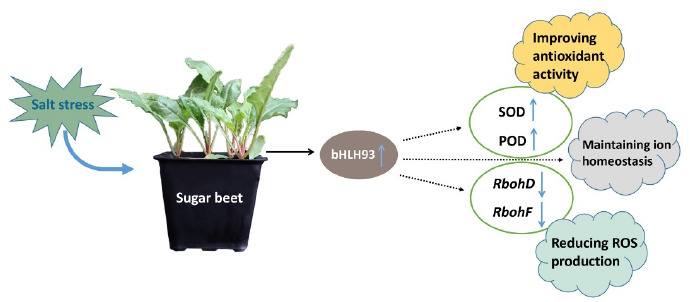
Overview diagram showing how the BvbHLH93 functions in mediating plant salt stress tolerance. Salt stress can turn on the expression of *BvbHLH93*, which plays an important role in activating ROS-detoxifying enzymes, leading to reduced ROS levels and maintaining ion homeostasis for plant salt stress tolerance.

Two plasma membrane localized NADPH-oxidases, RbohD and RbohF, were found to play key roles in the production of ROS, and the expression of *RbohD* and *RbohF* is often related to ROS generation [32,33]. In this study, expression of *RbohD* and *RbohF* was dramatically enhanced in the WT under salt stress, and the increased expression was inhibited by overexpressing the *BvbHLH93* in the transgenic plants (Figure 7). These results show that BvbHLH93 regulates salt stress tolerance by improving antioxidant activity and reducing ROS production (Figure 8). Recently, the overexpression of a *S-adenosylmethionine decarboxylase*, a key rate-limiting enzyme that participated in the biosynthesis of polyamines in sugar beet, also decreased expression of *RbohD* and *RbohF* and reduced cell membrane damage [34]. Therefore, whether *Bv*bHLH93 can decrease the expression of *RbohD* and *RbohF* through regulating polyamine metabolism needs to be further studied (Figure 8).

## 4. Materials and Methods

### 4.1. Plant Materials, Growth Conditions, and Salt Stress Treatment

Seeds of sugar beet T510 were obtained from Heilongjiang University. Sugar beet seeds were sown in vermiculite and watered daily. After one week, seedlings were transferred to 10 L hydroponic containers with Hoagland solution. Seedlings were grown in a quantum flux density of 450 μmol m^−2^ s^−1^ and incubated at 25 °C with a 14/10 h photoperiod in a greenhouse. Salt stress treatment (200 mM NaCl) was initiated three weeks after sowing, and the tissues of sugar beet were harvested and stored in −80 °C for subsequent experiments.

The Columbia (Col-0) wild type *Arabidopsis* was used to generate transgenic plants. *Arabidopsis* mutant seeds were obtained from TAIR (https://www.arabidopsis.org, accessed on 15 February 2018). The seeds were surface sterilized and incubated on Petri dishes containing Murashige and Skoog (MS) medium at 4 °C for three days before germination. Then, the seeds were germinated at 22 °C under 250 μmol m^−2^ s^−1^ light in a 14 h light/10 h darkness cycle. The eight-day-old seedlings were transferred to MS medium containing 150 mM NaCl for seven day salt treatment.

### 4.2. Cloning the Full-Length cDNA of BvbHLH93 and Sequence Analysis

Total RNA of sugar beet T510 leaves was extracted using a Trizol reagent following the manufacturer’s instructions (Invitrogen), and first-strand cDNA was synthesized by reverse transcription PCR Kit from TaKaRa. The full length cDNA sequence of *BvbHLH93* was obtained by PCR amplification with primer designed by Primer 5 (*BvbHLH93-F/R* (full length cDNA) in Table S1). Theoretical isoelectric points and molecular weights of the *BvbHLH93* protein sequence were determined using the ExPASy Compute pI/MW tool (https://web.expasy.org/compute_pi/, accessed on 10 February 2020). DNAMAN software was used for multiple sequence alignment. Phylogenetic tree of *Bv*bHLH93 was generated using MEGA5.0 and Clustal X2.0.

### 4.3. Subcellular Localization Analysis of BvbHLH93

To analyze the location of *Bv*bHLH93 protein, the ORF cDNA sequence of *BvbHLH93* was amplified by specific primers designed by Primer 5 (*BvbHLH93-F/R* (GFP vector construct) in Table S1) without a stop codon through the RT-PCR method. Then, the *BvbHLH93* was inserted into pCAMBIA2300-GFP with *EcoR*I/*Sal*I enzyme. The recombinant construct and empty vector were introduced into *Agrobacterium* GV3101 separately. Three-week-old leaves of *N. benthamiana* were infiltrated with *Agrobacterium* harboring the recombinant construct or empty vector [35]. DAPI for the staining of the nucleus was obtained from Life Technologies. *N. benthamiana* leaf epidermal cells were examined with a confocal laser scanning microscope (Olympus, Tokoyo, Japan).

### 4.4. Assay of BvbHLH93 Transcription Activation

The entire *BvbHLH93* coding sequence was obtained by PCR with the primers designed by Primer 5 (*BvbHLH93-F/R* (yeast vector construct) in Table S1). The PCR products were digested with *BamH*I and subcloned to pGBKT7 vector to generate pGBKT7-*BvbHLH93*. The plasmids of pGBKT7 (negative control) and pGBKT7-*BvbHLH93* were transformed into yeast *AH109*. The transformed yeast cells were plated on SD/-Trp and SD/-Trp-His-Ade growth media. The plates were observed after 3–5 days. If the BvbHLH93 protein has transcriptional activation activity, the transformant pGBKT7-BvBHLH93 can grow normally on SD-Trp-His-Ade medium.

### 4.5. Quantitative Real-Time PCR Analysis

Total RNA was extracted using the TRizol reagent. First-stand cDNA was synthesized by a PrimeScript First-strand cDNA Synthesis Kit (TaKaRa, Dalian, China). Real-time PCR was performed in a Two-color Real-time PCR Detection System (Bio-Rad, Hercules, CA, USA) with SYBR Green PCR Mix (TaKaRa, Dalian, China). The *18S rRNA* and *Actin* were chosen as internal controls in sugar beet and *Arabidopsis*, respectively [36]. PCR reaction was carried out in 10 µL volumes using the following amplification protocol: 94 °C for 4 min; 94 °C for 30 s, 53 °C for 20 s, and 72 °C for 70 s; and 72 °C for 4 min and 45 cycles. The primers used for qRT-PCR analysis are listed in Table S1. The primer sequence of *SOD* and *POD* genes for qRT-PCR were acquired from reference [30], and other primers were designed by Primer 5. A total of three biological replicates and three technical replicates were performed for the quantitative Real-Time PCR analyses.

### 4.6. Generation of BvbHLH93 Transgenic Arabidopsis Plants

To construct 35S: *BvbHLH93*, the full-length *BvbHLH93* coding sequence was amplified by PCR using gene-specific primers (*BvbHLH93-F/R* (overexpression vector construct) in Table S1), and the PCR products were digested with *Xba*I/*Sal*I and cloned into the pCAMBIA1300 vector. The constructs were introduced into *Agrobacterium* GV3101 and transformed into *Arabidopsis* plants through a floral dip method [37]. Transgenic plants were selected using 30 µg mL^−1^ hygromycin, and gene expression was confirmed by genotyping PCR and qRT-PCR. T3 homozygous transgenic lines were used for all experiments.

### 4.7. Measurements of Physiological Indicators

Chlorophyll (a+b) content was detected using our previously reported method [38]. Fresh leaves of 0.1 g were homogenized in liquid nitrogen, followed by addition of 1.5 mL of 80% acetone. Then, the mixture was incubated in the dark for 1.5 h, and centrifuged at 16,000 rpm for 4 min. Absorbance of the extract was determined at 663 and 645 nm [38]. For Na^+^ and K^+^ analysis, dried leaf tissue was ground and passed through a 2 mm mesh sieve. A 0.5 g weighed sample was used, and Na^+^ and K^+^ contents were determined using a flame atomic spectrophotometer [39]. Malondialdehyde (MDA) content was measured by the thiobarbituric acid (TBA) reaction using the method described by Chołuja et al. [40]. For the antioxidant enzyme assays, SOD and POD activities were measured as previously published by our laboratory [34,41].

### 4.8. Statistical Analysis

For all the experiments, three biological replicates with three technical replicates of each treatment were measured. All data were analyzed using GraphPad Prism 6 LSD method, and subjected to one-way ANOVA of SPSS (Statistical Product and Service Solutions) for testing significance, with *p* < 0.05 as cutoff for significant differences.

## Data Availability

Not applicable.

## Supplementary Materials

The following are available online at https://www.mdpi.com/article/10.3390/ijms22073669/s1: Figure S1: Sequence analysis of a cDNA encoding a *BvbHLH93* isolated from the sugar beet. Nucleotide and deduced amino acid sequence of the BvbHLH93. Figure S2: Identification of *atbhlh93* mutant and overexpression of *BvbHLH93* in *Arabidopsis*. Table S1: The list of primers used in this article.
