# Supplementary material for: Functional Characterization of a Sugar Beet BvbHLH93 Transcription Factor in Salt Stress Tolerance
Source: Int J Mol Sci. 2021 Apr 1;22(7):3669. doi: 10.3390/ijms22073669 (PMC8037259; doi:10.3390/ijms22073669)
Supplement: Supplementary file 1 [file ijms-22-03669-s001.pdf]

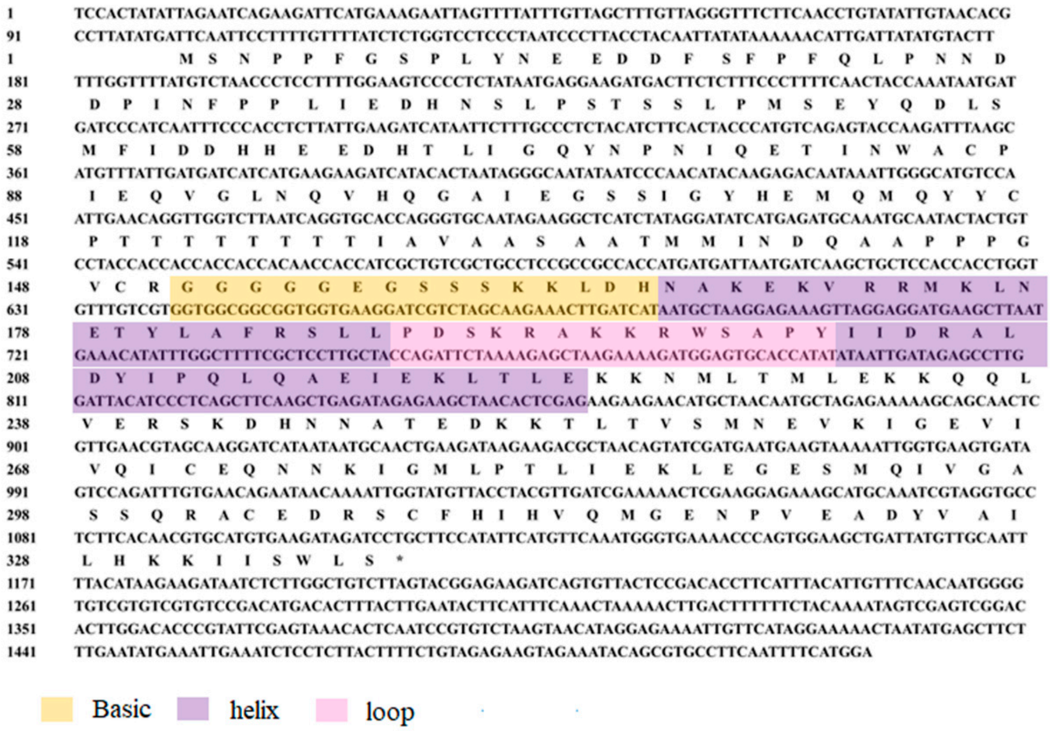

Figure S1. Sequence analysis of a cDNA encoding a *BvbHLH93* isolated from the sugar beet. Nucleotide and deduced amino acid sequence of the *BvbHLH93*.

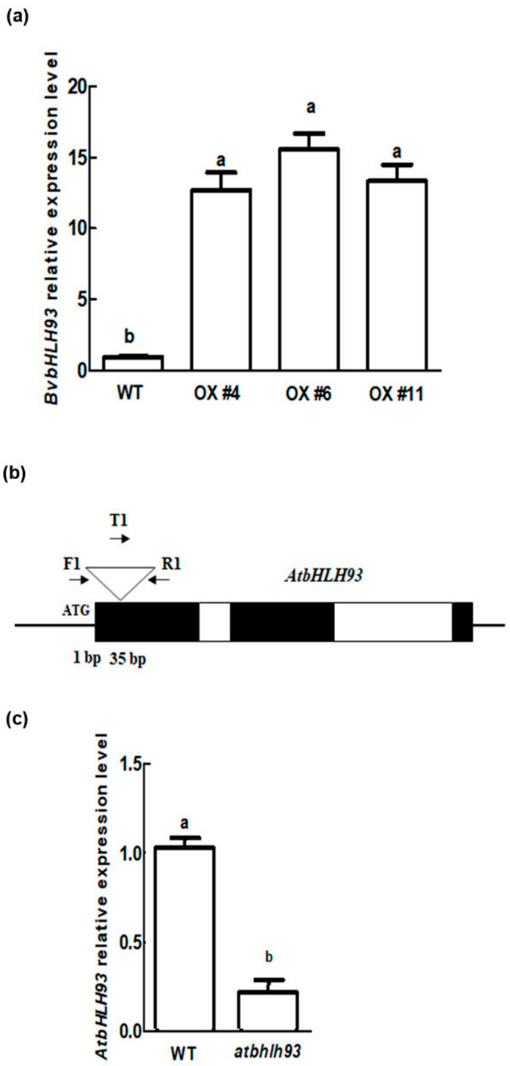

Figure S2. Identification of *atbhlh93* mutant and overexpression of *BvbHLH93* in *Arabidopsis*. (a) QRT-PCR analysis of the expression levels of the overexpressed *BvbHLH93* (OX4, OX6 and OX11) in *Arabidopsis*; (b) Structure of the *AtbHLH93* gene. The T-DNA insertion site was at 35 bp downstream of the start codon. The primers used to identify the T-DNA insertion were marked with arrows (T1, R1 and F1); (c) Real-time PCR analysis of the expression levels of *AtbHLH93* in the *atbhlh93* mutant. Different letters indicate significant difference at $p < 0.05$. Three biological replicates were performed. Please refer to supplementary materials for the primers used.

Table S1. The list of primers used in this article

Primer name	Primer sequence
BvbHLH93-F (Full length cDNA)	5-TCCACTATATTAGAATCAGAAGATT-3
BvbHLH93-R (Full length cDNA)	5-TCCATGAAAATTGAAGGCACGCTGT-3
BvbHLH93-F (GFP vector construct)	5-CCGGAATTCATGTCTAACCCTCCTTTTGG-3
BvbHLH93-R (GFP vector construct)	5-ACGCGTCGACAGCCAAGAGATTATCTTC-3
BvbHLH93-F (Yeast vector construct)	5-GAATTCCCGGGGATCATGTCTAACCCTTTTGG-3
BvbHLH93-R (Yeast vector construct)	5-GCAGGTCGACGGATCCTAAGACAGCCAAGAGATTA-3
BvbHLH93-F (Overexpression vector construct)	5-CTAGTCTAGAATGTCTAACCCTCCTTTTGG-3
BvbHLH93-R (Overexpression vector construct)	5-ACGCGTCGACAGACAGCCAAGAGTCGAC-3
BvbHLH93-F (QRT-PCR)	5- AAGCAGCAACTCGTTGAACG-3
BvbHLH93-R (QRT-PCR)	5- CATGCACGTTGTGAAGAGGC-3
Bv18S rRNA-F (QRT-PCR)	5-CCCCAATGGATCCTCGTTA-3
Bv18S rRNA-R (QRT-PCR)	5-TGACGGAGAATTAGGGTTCG-3
AtRbohD (At5g47910)-F (QRT-PCR)	5-TCAGGGACGACTCGGTGG-3
AtRbohD (At5g47910)-R (QRT-PCR)	5-GTTTATCGAAACGTTGGTC-3
AtRbohF (At1g64060)-F (QRT-PCR)	5-GTTCGATGCATTGAGTAG-3
AtRbohF (At1g64060)-R (QRT-PCR)	5-TTTAATCTTGATAGCTTATT-3
AtbHLH93 (At1g71200)-F (QRT-PCR)	5-TGGCTCAAGTAGTGGTGCAG-3
AtbHLH93 (At1g71200)-R (QRT-PCR)	5-CCCCCAATTCAAGAACCGAGA-3
β-Actin (At3g53750)-F (QRT-PCR)	5- GAGGCTCCTCTTAACCCAA-3
β-Actin (At3g53750)-R (QRT-PCR)	5-TACAATTTCCCGCTCTGC-3
SOD (At5g18100)-F (QRT-PCR)	5-AACGAGGAAGAGCGTCATG-3
SOD (At5g18100)-R (QRT-PCR)	5-GCGTTTCCAGTTGATTTG-3
SOD (At4g25100)-F (QRT-PCR)	5-ATGAGAAGTTCTATGAAGAG-3
SOD (At4g25100)-R (QRT-PCR)	5-GTCTTTATGTAATCTGGT-3
POD (At1g14550)-F (QRT-PCR)	5-CCATAGGACAATCTCAATGC-3
POD (At1g14550)-R (QRT-PCR)	5-TGATCGGTTACTAATAG-3

POD (At5g66390)-F （QRT-PCR）	5-CTCACTAAGTTCAAGCGTC-3
POD (At5g66390)-R （QRT-PCR）	5-GAATAGGGTCTGGTCACC-3
